# Immediate Psychological Effects of the COVID-19 Quarantine in Youth From Italy and Spain

**DOI:** 10.3389/fpsyg.2020.579038

**Published:** 2020-11-06

**Authors:** Mireia Orgilés, Alexandra Morales, Elisa Delvecchio, Claudia Mazzeschi, José P. Espada

**Affiliations:** ^1^Department of Health Psychology, Universidad Miguel Hernández, Elche, Alicante, Spain; ^2^Department of Philosophy, Social Sciences and Education, Università degli Studi di Perugia, Perugia, Italy

**Keywords:** COVID-19, quarantine, emotional impact, habits, youths

## Abstract

The COVID-19 quarantine has affected more than 860 million children and adolescents worldwide, but to date, no study has been developed within Western countries to examine the psychological impact on their lives. The present study aims to examine for the first time the emotional impact of the quarantine on children and adolescents from Italy and Spain, two of the countries most affected by COVID-19. Participants were 1,143 parents of Italian and Spanish children aged 3 to 18 years who completed a survey providing information about how the quarantine affects their children and themselves, compared to before the home confinement. Results show that 85.7% of the parents perceived changes in their children’s emotional state and behaviors during the quarantine. The most frequent symptoms were difficulty concentrating (76.6%), boredom (52%), irritability (39%), restlessness (38.8%), nervousness (38%), feelings of loneliness (31.3%), uneasiness (30.4%), and worries (30.1%). Spanish parents reported more symptoms than Italians. As expected, children of both countries used monitors more frequently, spent less time doing physical activity, and slept more hours during the quarantine. Furthermore, when family coexistence during quarantine became more difficult, the situation was more serious, and the level of stress was higher, parents tended to report more emotional problems in their children. The quarantine impacts considerably on Italian and Spanish youth, reinforcing the need to detect children with problems as early as possible to improve their psychological well-being.

## Introduction

The outbreak of the 2019 coronavirus disease (COVID-19) emerged in the city of Wuhan (China) in December 2019. Motivated by its rapid spreading, the World Health Organization (WHO) declared it a pandemic on March 11, 2020 (World Health Organization (WHO), 2020). Italy and Spain are two of the most affected countries worldwide, with, respectively, more than 29,000 and 25,000 reported deaths as of May 1 (European Centre for Disease Prevention and Control, 2020) since its spread. Spain has the world’s second-highest number of deaths in relation to the number of inhabitants, with 544 deaths per million inhabitants, followed by Italy, with 481 deaths per million inhabitants, as of May 5 (European Centre for Disease Prevention and Control, 2020). Following the Chinese government’s retarding the spread of COVID-19, quarantine was implemented in Italy and Spain, among other countries, starting on March 10 in Italy and 6 days later in Spain. School closure was mandatory, and gathering in public spaces, with certain exceptions, was prohibited. During school closures, children’s routines change, and healthy behaviors, such as physical activity, adequate diet, or good sleep habits, could be less probable to happen (Brazendale et al., 2017). Also, as a recent review of studies concludes, the limited social interaction increases loneliness, which is associated with mental health problems in children and adolescents (Loades et al., 2020). However, the effect of closing schools as a measure to decrease virus transmission is inconclusive. According to a systematic review of studies focused on other previous coronavirus outbreaks, school closure did not seem to have a very determining effect compared to other social distancing measures, and following some COVID-19 modeling studies, school closure may only reduce between 2 and 4% of deaths (Viner et al., 2020). Esposito and Principi (2020) highlighted alternative strategies, such as reducing class size or physical distancing, considering the adverse effects of school closure on children and their families (e.g., the economic consequences for parents who remain at home to take care of their children, or problems in implementing distance learning in the poorest areas of some countries). However, a recent study examining parents of Czechia children show that families tend to cope well with education at home during the COVID-19 confinement, although they consider that children would need more time for learning activities (Brom et al., 2020).

This is the first time that a quarantine to control a pandemic has been implemented in Italy and Spain, as well as in most countries worldwide. Therefore, there is a lack of conclusive studies providing data on how this measure can affect children and adolescents. Regarding the specific psychological consequences of COVID-19 and the measures to cope with them, few studies have been published. The psychological impact on the Chinese population after 2 weeks of quarantine was rated as moderate or brief by 53.8% of the 1,210 participants in a study with adults from 194 cities in China (Wang C. et al., 2020). Depression symptoms of moderate to severe severity were reported by 16.5% of participants, anxiety by 28.8%, and stress by 8.1%. The results of a study with 4,607 adults aged 17 to 90 years from 31 regions of China, however, reported slight changes in the frequency with which participants experienced negative and positive emotions before and after the COVID-19 imposed quarantine. Rather, some benefits were found, with reports of fewer sleep problems, fewer aggressive behaviors, and less alcohol and tobacco use during the quarantine. As the authors of the study point out, among other reasons, the quarantine was imposed just before the Chinese New Year holidays, when the majority of the population were at home with their families, thus having social support to reduce stress (Li et al., 2020). Concerning the specific stressors affecting the population during the quarantine, a review of 24 studies highlights as main stressors the duration of the quarantine, the fear of infection, frustration and boredom, and not having adequate information or clear guidelines from public authorities; as main stressors after the quarantine, having financial problems and the stigma for people who were infected or exposed to the disease were reported (Brooks et al., 2020).

To date, no studies have examined how the quarantine declared due to COVID-19 may affect children’s and adolescents’ emotional or behavioral well-being within Western countries. Some previous studies suggest that the effects may be troublesome. In a recently published study, 23% of Chinese school-age children reported depressive symptoms, and 19% reported anxiety symptoms after 34 days of COVID-19 confinement (Xie et al., 2020). A review of 190 studies with an American population concluded that, compared to vacation periods and weekends, by being more structured, class days give children more opportunities to be physically active, spend less time in front of screens, and regulate their sleep schedules (Brazendale et al., 2017). Post-traumatic stress is estimated to be four times higher in children who have been in quarantine compared to those who have not, and their likelihood of presenting acute stress disorder, adjustment disorder, and grief is also higher (Sprang and Silman, 2013). Among the possible consequences of the COVID-19 emergency, a main concern is suicidal ideation, as stressful life events are considered a psychosocial risk factor for suicidality (Carballo et al., 2020). Some authors have reflected on the possible effects of the COVID-19 quarantine on children and adolescents. Wang G. et al. (2020) highlight the need for awareness of the quarantine’s potential effects on children’s mental health and the importance for governments, non-governmental organizations, the community, schools, and parents to act to reduce the possible effects of this situation. Special attention should be paid to children and adolescents who are separated from their caregivers who are infected or suspected of being infected and those whose caregivers are infected or have died, because they are more vulnerable to psychological problems (Liu et al., 2020). It is very important to identify childhood mental health problems as soon as possible, differentiating normal and pathological reactions through the use of screening tools that may indicate the need for intervention (Espada et al., 2020; Liu et al., 2020).

COVID-19 confinement changed the lives of most children and adolescents. Social relationships and academic routines were changed by virtual friends and distance learning; leisure was restricted to indoors as public spaces were closed. Italy and Spain had one of the most restrictive home confinement rules, not allowing children to go outside until 3 and 6 weeks, respectively, after the start of confinement. Although confinement was necessary to break the pandemic, the interruption of all social contact and the prohibition from going outside home could have had immediate effects in children and adolescents. Controversy in both countries arose over whether confinement would affect children or whether they could adapt to the new situation without being emotionally affected. Knowing if confinement has effects on the well-being of children would help professionals to implement preventive measures and governments, less strict confinement rules. Despite that, so far, we have not found any study that examines the effect in children and adolescents of the quarantine imposed by COVID-19 within Western countries. The available studies have been carried out with adult populations—hence, the results cannot be extrapolated to child populations—and with Chinese populations, whose cultural differences with the West make it difficult to generalize their findings. This study is the first to determine the immediate psychological responses in children and adolescents of the West to the quarantine imposed to put an end to COVID-19. The main objective of the study is to examine the emotional well-being of Italian and Spanish children aged between 3 and 18 who are in quarantine as a measure imposed by governments to prevent the transmission of COVID-19. Specifically, the aim is to know: (a) the immediate psychological responses in children and adolescents during the quarantine perceived by parents, (b) the emotional impact of the quarantine on children’s primary caregivers, (c) the relationship between the parents’ emotional state and their children’s immediate psychological responses, (d) the change in childrens, habits, and (e) the relationship between the parents’ emotional state and the change in their children’s habits. According to the only study carried out to date to examine the immediate effects of COVID-19 in children (Xie et al., 2020), and to findings with adult samples (e.g., Wang C. et al., 2020), it is expected that confinement affects the well-being of Italian and Spanish children, as it affected the Chinese population.

## Materials and Methods

### Participants

Table 1 reports sample characteristics and differences in sociodemographic variables by country. Of the 1,143 participants, 62.3% were recruited from 94 cities in Italy, and the rest, from 87 cities in Spain. The respondents were aged 18 to 66 and were the primary caregiver of children aged 3 to 18 (47.5% were females). Most children were not diagnosed with a physical or psychological problem (89%). The caregivers’ educational level was relatively high; more than half of them (61.9%) had attended college to earn an undergraduate degree, a Master’s degree, or a Ph.D., and only 6.9% had basic studies. The mothers’ and fathers’ current employment situations varied. In many cases, the parents had a full-time job or smart-worked. Around 5% reported having lost their job because of the COVID-19 situation. More than half of the participants (69.4%) reported having some at-risk friends or family, but they did not live with them during the quarantine; only 9% belonged to an at-risk group.

**TABLE 1:** Sample characteristics and differences by country.

	**Total (*n* = 1,143)**	**Italy (*n* = 712)**	**Spain (*n* = 431)**	**Test^a^**	***z***	***p*^b^**	**Effect size^c^**
**Parents**							
Female, *N* (%)	1,006(88)	627 (88.1)	379 (87.9)	0.004		0.94	–
Age, *M* (*SD*)	42.30 (6.17)	42.38 (6.64)	42.17 (5.32)	14,870	−1.21	0.22	–
**Educational level, *N* (%)**							
Doctoral or Master’s degree	213 (18.6)	107 (15)	106 (24.6)	48.89		<0.001	0.20
Undergraduate	495 (43.3)	297 (41.7)	198 (45.9)				
Secondary school	356 (31.1)	270 (37.9)	86 (20)				
Primary school	79 (6.7)	38 (5.4)	41 (9.5)				
**Monthly family income (euros)**							
Up to 999	64 (6.4)	33 (5.3)	31 (8.3)	9.03		0.06	–
Between 1,000 and 1,999	277 (27.7)	164 (26.2)	113 (30.1)				
Between 2,000 and 2,999	307 (30.7)	209 (33.4)	98 (26.1)				
Between 3,000 and 4,999	275 (27.5)	169 (27)	106 (28.3)				
5,000 or more	78 (7.7)	51 (8.1)	27 (7.2)				
**Mother’s current employment situation**							
Self-employed	180 (16)	118 (16.8)	62 (14.5)	55.93		<0.001	0.22
Part-time	168 (14.9)	132 (18.9)	36 (8.4)				
Full-time	285 (25.2)	176 (25.2)	109 (25.5)				
Unemployed	103 (9.1)	36 (5.1)	67 (15.7)				
Lost job due to COVID-19	57 (5.1)	30 (4.3)	27 (6.3)				
Smart-working	260 (23.1)	157 (22.5)	103 (24.1)				
Other	74 (6.6)	50 (7.2)	24 (5.5)				
**Father’s current employment situation**							
Self-employed	279 (25)	184 (26.6)	95 (22.4)	43.31		<0.001	0.20
Part-time	28 (2.5)	15 (2.2)	13 (3.1)				
Full-time	503 (45.1)	335 (48.5)	168 (39.5)				
Unemployed	32 (2.9)	15 (2.2)	17 (4)				
Lost job due to COVID-19	51 (4.6)	17 (2.5)	34 (8)				
Smart-working	200 (17.9)	120 (17.4)	80 (18.8)				
Other	22 (2)	4 (0.6)	18 (4.2)				
**Your situation concerning COVID-19**							
I belong to a risk group	103 (9)	41 (5.8)	62 (14.4)	34.07		<0.001	0.17
People belonging to a risk group live with me	151 (13.2)	94 (13.2)	57 (13.2)				
Friends or family are at-risk population (not living with us)	793 (69.4)	501 (70.4)	292 (67.7)				
I do not know anyone who belongs to an at-risk population	96 (8.4)	76 (10.6)	20 (4.7)				
**The house where you live has, *N* (%)**							
Only windows	102 (8.9)	25 (3.5)	77 (17.9)	167.97		<0.001	0.38
Garden	445 (38.9)	368 (51.7)	77 (17.9)				
Terrace	272 (23.8)	151 (21.1)	121 (28.1)				
Balcony	286 (25)	141 (19.9)	145 (33.5)				
Another exit	38 (3.3)	27 (3.8)	11 (2.6)				
Square meters home, *M* (*SD*)	126.11 (63.22)	123.14 (62.29)	124.99 (62.86)	136,342	−3.31	0.001	0.09
**Children**							
Female, *N* (%)	543 (47.5)	351 (49.3)	192 (44.5)	2.42		0.11	–
Age, *M* (*SD*)	9.08 (4.22)	9.40 (4.46)	8.55 (3.73)	138,750.50	−2.72	0.006	0.08
Physical or psychological problems. Yes, *N* (%)	126 (11)	52 (7.3)	74 (17.2)	26.64		<0.001	0.15
Is your child receiving treatment for that problem? Yes, *N* (%)	96 (8.4)	42 (5.9)	54 (12.5)	45.32		<0.001	0.19

*M, mean; *SD*, standard deviation. ^*a*^*χ*^2^ for categorical variables and *U* Mann–Whitney for continuous variables. ^*b*^ Bonferroni correction applied to *p* values was used to reduce the risk of type I errors of a chi-squared test. ^*c*^ Effect size = Cramer’s *V* for multi-categorical variables and Rosenthal *r* statistic for continuous variables.*

The Italian and Spanish samples were equivalent, except for respondents’ educational level, the mothers’ or fathers’ current employment situation, respondents’ situation concerning COVID-19, children’s age, and the proportion of children with physical or psychological problems (Table 1). In the Spanish sample, there was a higher proportion of parents with postgraduate or doctoral studies or with secondary education than in the Italian sample. Compared to Spain, a higher proportion of Italian women were employed (84.3 vs. 94.8%), and they had part-time jobs (8.4 vs. 18.9%). The Italian children were slightly older than the Spanish ones (9.40 vs. 8.55 years), although the effect size was very low.

### Procedure

Participants were recruited via social networks (Twitter, Facebook, WhatsApp, Instagram), as face-to-face contact was not allowed. An online survey was created *ad hoc* using Google Forms and distributed in each country using a snowball sampling strategy. Before participants completed the survey, information about the objectives of the study was provided, and informed consent was requested. Data were collected for 15 days in both countries, with the study starting 15 days after the lockdown. The approval of the Ethics Board of the authors’ institution was obtained for the research.

### Survey Development

Scientific literature related to the psychological impact of quarantine was reviewed by six experts in clinical psychology, and questionnaires applied in previous studies with adult populations were considered for creating the survey. After a pilot study with a group of parents was conducted, the final version of the questionnaire was structured in four sections, collecting information on: (a) the sociodemographics of parents and children (see Table 1); (b) parental perception on how quarantine emotionally affects children through 31 symptoms, ranging from 1 (*much less compared to before quarantine*) to 5 (*much more compared to before quarantine*), reaching a Cronbach’s alpha of 0.95; (c) parents’ perception of family coexistence during quarantine, severity of the situation caused by coronavirus with regard to the family’s well-being, and parents’ stress, on a five-point scale; and (d) children’s routines: time of screen use, physical activity, and hours of sleep during quarantine compared to before.

### Data Analyses

All analyses were performed with SPSS v.26 for Mac. Descriptive statistics were run to analyze participants’ sociodemographic variables and other variables of interest for the study. Because the variables were not normally distributed (according to the Kolmogorov–Smirnov test, *p* < 0.05), non-parametric tests were used. Differences between Italy and Spain in sociodemographic variables, children’s psychological responses, emotional impact on the children’s primary caregivers, and the children’s routines were analyzed using chi-square (χ^2^) (categorical variables) and the Mann–Whitney *U* test (continuous variables). Bonferroni corrections applied to *p* values were used to reduce the risk of type I error. For example, considering α = 0.05 and the comparison of 31 symptoms between both countries, adjusted alpha was set at 0.0016. The odds ratio (OR) was reported for 2 × 2 tables. The effect size of the intergroup differences was calculated using the Rosenthal *r* statistic, which is interpreted according to the following ranges: 0.1, small; 0.3 medium; and 0.5, large (Rosenthal, 1991). Cramer’s *V* was calculated as a measure of association between multi-categorical variables and interpreted as follows: >0.25, very strong; >0.15, strong; >0.10, moderate; >0.05, weak; and >0, none or very weak (Akoglu, 2018). Multivariate analyses were performed by generalized linear (GENLIN) modeling. For each child psychological reaction, GENLIN modeling was used to examine differences between both countries, adjusting for baseline differences, parents’ age, and parental stress. GENLIN models for child routines were used to identify changes during home confinement, compared to before this period and between both countries during home confinement. All models were adjusted for baseline differences between both countries, parents’ age, and parental stress (since this variable was the most related to child psychological reactions). All models were adjusted for sociodemographic differences between countries. Because of the ordinal nature of the variables, Spearman correlations were calculated to analyze the relationship between the primary caregivers’ emotional effects due to COVID-19 and their children’s psychological responses. The relationship between changes in children’s habits (time of screen use, physical activity, and hours of sleep) and parental variables (parents’ perception of family coexistence during quarantine, severity of the situation caused by coronavirus with regard to the family’s well-being, and parents’ stress) was also analyzed using Spearman correlations. A new variable, “change,” was created by subtracting the score on the child routine variables at the time “before confinement” from that “during quarantine.”

## Results

### Parental Perception of the Emotional Effects of the Quarantine on Their Children

Nine hundred and eighty parents (85; 83.8 in Italy and 88.9% in Spain) observed changes in their children’s emotional state and behaviors during the quarantine. The most common changes (present in at least 20% of the responses) were that, during quarantine, their children had more difficulty concentrating (76.6%), felt more bored than usual (52%), were more irritable (39%), were more restless (38.8%), were more nervous (38%), felt lonelier (31.3%), were more uneasy (30.4%), were more worried (30.1%), were more likely to argue with the rest of the family (29.7%), were more dependent on them (28%), were more anxious (28.4%), were angrier (25.9%), were more reluctant (24.7%), were sadder (23.3%), were afraid of COVID-19 infection (23.1%), were more worried when someone left the house (22%), and ate more than usual (21.9%). Table 2 details the percentage of primary caregivers who perceived changes in their children’s emotional state and behaviors during the quarantine and the differences between Italy and Spain.

**TABLE 2:** Primary caregivers’ perception of the emotional and behavioral effects of the quarantine in their children.

	**Total**		**Italy**		**Spain**		**β**	**SE**	**Wald χ^2^ 95% CI**
	***N***	**%**	***n***	**%**	***n***	**%**			
My child is worried	344	30.1	226	31.7	118	27.4	0.20	0.14	−0.08, 0.49
My child is restless	443	38.8	247	34.7	196	45.5	0.38	0.13	0.12, 0.65
My child is anxious	325	28.4	146	20.5	179	15.7	1.02	0.14	0.73, 1.31***
My child is sad	266	23.3	189	26.5	77	17.9	−0.61	0.16	−0.93, −0.29***
My child has nightmares	126	11	62	8.7	64	14.8	0.39	0.20	−3.73, 0.79
My child is reluctant	282	24.7	192	27	90	20.9	−0.32	0.15	−0.63, −0.01
My child feels lonely	358	31.3	280	39.3	78	18.1	−1.14	0.15	−1.45, −0.83***
My child wakes up frequently	138	12.1	70	9.8	68	15.8	0.38	0.19	0, 0.77
My child sleeps little	125	10.9	52	7.3	31.3	16.9	0.91	0.21	0.50, 1.32***
My child is very indecisive	131	11.5	62	8.7	69	16	0.54	0.21	0.12, 0.95
My child is uneasy	347	30.4	184	25.8	163	37.8	0.45	0.14	0.17, 0.73***
My child is nervous	434	38	243	34.1	191	44.3	0.38	0.13	0.11, 0.65
My child is afraid to sleep alone	197	17.2	94	13.2	103	23.9	0.62	0.17	0.28, 0.97***
My child argues with the rest of the family	339	29.7	165	23.2	174	40.4	0.86	0.14	0.58, 1.15***
My child is very quiet	126	11	102	14.3	24	5.6	−0.85	0.25	−1.35, −0.35***
My child cries easily	195	17.1	97	13.6	98	22.7	0.50	0.17	0.16, 0.85
My child is angry	296	25.9	157	22.1	139	32.3	0.47	0.14	0.18, 0.77***
My child asks about death	155	13.6	102	14.3	53	12.3	−0.49	0.20	−0.90, −0.09
My child feels frustrated	213	18.6	113	15.9	100	23.2	0.35	0.16	0.02, 0.68
My child is bored	596	52.1	383	53.8	213	49.4	−0.21	0.13	−0.47, 0.04
My child is irritable	446	39	260	36.5	186	43.2	0.20	0.13	−0.06, 0.46
My child has sleeping difficulties	195	17.1	90	7.9	105	9.2	0.64	0.17	0.30, 0.98***
My child has no appetite	98	8.6	48	6.7	50	11.6	0.54	0.23	0.07, 1.01
My child is easily alarmed	138	12.1	78	11	60	13.9	0.11	0.20	−0.28, 0.52
My child has difficulty concentrating	875	76.6	577	81	298	69.1	0.59	0.15	0.30, 0.89***
My child is afraid of COVID-19 infection	264	23.1	164	23	100	23.2	−0.06	0.15	0.37, 0.23
My child is very dependent on us	320	28	163	22.9	157	36.4	0.56	0.14	0.27, 0.85***
My child has physical complaints (headache, stomach ache.)	159	13.9	72	10.1	87	20.2	0.68	0.18	0.32, 1.05***
My child has behavioral problems	185	16.2	57	8	128	29.7	1.46	0.18	1.10, 1.83***
My child eats a lot	250	21.9	142	19.9	108	25.1	0.22	0.15	−0.08, 0.52
My child worries when one of us leaves the house	251	22	121	17	130	30.2	0.69	0.15	0.38, 0.99***

χ^2^ = chi-square; OR = odds ratio; CI = confidence interval. ***p < 0.001. Bonferroni correction applied to *p* values was used to reduce the risk of type I errors of a chi-squared test.

Spanish children were significantly more psychologically affected than Italian children during the quarantine (88.9 vs. 83.8%; β = 0.38, SE = 0.19, Wald χ^2^ 95% CI [0, 0.77], *p* = 0.05), controlling for sociodemographic differences between samples from Italy and Spain, parents’ age, and parental stress. Compared to Italian children, Spanish children had more behavioral problems (8 vs. 29.7%), were more likely to argue with the rest of the family (23.2 vs. 40.4%), had more physical complaints (10.1 vs. 20.2%), were more afraid to sleep alone (13.2 vs. 23.9%), and were more worried when one of the parents left the house (for example, to buy groceries) (17 vs. 30.2%). Other differences between Spanish and Italian children’s emotional state and behaviors during the quarantine are detailed in Table 2. Compared to Spanish children, Italians felt sadder (17.9 vs. 26.5%) and lonelier (18.1 vs. 39.3%) during the quarantine.

### Family Coexistence During the Quarantine, Perception of Severity, and Stress Due to COVID-19

Primary caregivers reported that family coexistence during the quarantine was found to be moderately easy (*M* = 3.68, *SD* = 1.98, range = 1–5). Only 11.4% reported that family coexistence during the quarantine was difficult or very difficult, and more than half (61.8%) informed that family coexistence was easy or very easy. The neutral option was selected by 26.8%. The parents perceived the current situation due to COVID-19 to be quite serious (*M* = 3.63, *SD* = 0.96, range = 1–5). Approximately one-half (55.4%) perceived the situation to be serious or very serious. Only 11.6% considered the current situation to be a little serious or not serious. The rest (33%) perceived the situation to be somewhat serious. Parental level of stress was moderate (*M* = 3.18, *SD* = 1.02, range = 1–5). Approximately one-third of the parents (35.4%) reported being stressed or very stressed, and 39.4% chose the option “somewhat stressed.” One in four (25.2%) parents indicated that they did not feel stressed because of the current situation.

When comparing the data of Italy and Spain, adjusting for differences in sociodemographic variables between both countries, parents’ age, and parental stress, no differences were observed in the perception of how easy it is for the family to live together during the quarantine (β = 0.04, SE = 0.06, Wald χ^2^ 95% CI [−0.07, 0.16], *p* = 0.43), how serious the current situation caused by the coronavirus is with regard to their well-being and their family’s well-being (β = 0.04, SE = 0.05, Wald χ^2^ 95% CI [−0.06, 0.15], *p* = 0.41), and the level of parental stress (β = 0.02, SE = 0.06, Wald χ^2^ 95% CI [−0.10, 0.14], *p* = 0.72).

### Relationship Between Primary Caregivers’ Perception of COVID-19 and Their Children’s Immediate Psychological Responses During the Quarantine

The primary caregivers’ perception of how easy it is for the family to live together during the quarantine was related to 11 of the 31 child symptoms (Table 3). When family coexistence during the quarantine was rated as more difficult, the parents tended to rate their children as more restless, more anxious and uneasy, more nervous, more likely to argue with the rest of the family, angrier, more frustrated, more irritable, having more difficulty concentrating, presenting more behavioral problems, and less likely to be quiet (compared to before home confinement). Spearman correlations were indirect (except for being quiet) and low, ranging from −0.06 to −0.15.

**TABLE 3:** Spearman correlations between primary caregivers’ perception of emotional and behavioral effects on their children and their perception of the situation due to COVID-19.

**Child symptoms**	**How easy is living together in the family?**	**How serious do you perceive the current situation to be?**	**How stressed do you feel?**
My child is worried	−0.003	0.124**	0.126**
My child is restless	−0.098**	0.109**	0.235**
My child is anxious	−0.063*	0.158**	0.238**
My child is sad	−0.017	0.060*	0.155**
My child has nightmares	−0.017	−0.010	0.019
My child is reluctant	−0.050	0.052	0.118**
My child is feels lonely	0.004	0.074*	0.099**
My child wakes up frequently	0.011	0.054	0.094**
My child is sleeps little	−0.011	0.051	0.046
My child is very indecisive	−0.022	0.053	0.116**
My child is uneasy	−0.078**	0.072*	0.200**
My child is nervous	−0.120**	0.110**	0.260**
My child is afraid to sleep alone	−0.026	0.048	0.106**
My child argues with the rest of the family	−0.155**	0.071*	0.188**
My child is very quiet	0.069*	−0.025	−0.063*
My child is cries easily	−0.032	0.061*	0.089**
My child is angry	−0.107**	0.100**	0.196**
My child asks about death	0.001	0.003	0.047
My child is feels frustrated	−0.084**	0.028	0.092**
My child is bored	−0.016	0.137**	0.183**
My child is irritable	−0.133**	0.066*	0.174**
My child has sleeping difficulties	−0.038	0.039	0.075*
My child has no appetite	0.022	0.007	0.002
My child is easily alarmed	−0.042	0.046	0.106**
My child has difficulty concentrating	−0.076**	0.073*	0.139**
My child is afraid of COVID-19 infection	0.040	0.140**	0.091**
My child is very dependent on us	−0.057	0.057	0.129**
My child has physical complaints (headache, stomach ache.)	−0.041	−0.001	0.036
My child has behavioral problems	−0.139**	0.025	0.102**
My child eats a lot	−0.021	0.048	0.042
My child worries when one of us leaves the house	−0.009	0.089**	0.074*

*p < 0.05; **p < 0.01.

Primary caregivers’ perception of the seriousness of the current situation caused by the coronavirus with regard to their well-being and their family’s well-being was associated with 15 of the 31 child symptoms. Caregivers who perceived the current situation as more serious with regard to their family’s well-being tended to report that, during quarantine, their children were more concerned, were more restless, were more anxious, were sadder, were lonelier, were more nervous and uneasy, were more likely to argue with the rest of the family, cried more easily, were angrier, were more bored, had more difficulty concentrating, were more afraid of COVID-19 infection, and were more worried when one of them left the house (compared to before home confinement). Spearman correlations were direct and low, ranging from 0.06 to 0.15.

Primary caregivers’ level of stress was related to 25 of the 31 child symptoms. Parents who perceived themselves as more stressed by the situation tended to report that, during quarantine, their children were more worried, were more restless, were more anxious, were sadder, were more reluctant, were lonelier, woke up more frequently, were more indecisive, were more uneasy, were more nervous, were more afraid to sleep alone, were more likely to argue with the rest of the family, cried more easily, were angrier, were more frustrated, were more bored, were more irritable, had more difficulty concentrating and sleeping, were more easily alarmed, were more afraid of COVID-19 infection, were more dependent on them, had more behavioral problems, and were more worried when one of them left the house (compared to before home confinement). Spearman correlations were direct, ranging from low (ρ = 0.07) to moderate (ρ = 0.26). However, parental stress was indirectly related to being quiet, suggesting that parents who are more stressed perceive their children as being less quiet, although this correlation was low (ρ = −0.06).

### Children’s Patterns of Use of Screens, Daily Physical Activity, and Hours of Sleep Before and During the Quarantine

Table 4 indicates that, during the quarantine, children spent more time daily using screens such as iPads, TVs, mobiles, or computers (β = 64.98, SE = 0.08, Wald χ^2^ 95% CI [55.38, 76.25], *p* < 0.001); spent less time doing physical activity (β = 0.04, SE = 0.07, Wald χ^2^ 95% CI [0.03, 0.04], *p* < 0.001); and tended to sleep a bit more (mean hours) (β = 1.24, SE = 0.07, Wald χ^2^ 95% CI [1.07, 1.42], *p* < 0.01) compared to before this period and controlling for parental’ age and stress. These changes in children’s routines during the quarantine were confirmed in both countries.

**TABLE 4:** Children’s patterns of use of screens, daily physical activity, and hours of sleep before and during the quarantine.

	**Total**		**Italy**		**Spain**	
**Children’s activity patterns**	**Before quarantine**	**During quarantine**	**Before quarantine**	**During quarantine**	**Before quarantine**	**During quarantine**
**Use of screens (min)**						
Less than 30	253 (22.1)	39 (3.4)	129 (18.1)	32 (4.5)	124 (28.8)	7 (1.6)
From 30 to 60	403 (35.3)	136 (11.9)	252 (35.4)	99 (13.9)	151 (35)	37 (8.6)
From 60 to 90	262 (22.9)	208 (18.2)	173 (24.3)	135 (19)	89 (20.7)	73 (16.9)
From 90 to 120	125 (10.9)	214 (18.7)	86 (12.1)	129 (18.1)	39 (9)	85 (19.7)
From 120 to 180	63 (5.5)	205 (17.9)	47 (6.6)	101 (14.2)	16 (3.7)	104 (24.2)
More than 180	37 (3.3)	341 (29.9)	25 (3.5)	216 (30.3)	12 (2.8)	125 (29)
**Physical activity (min per day)**						
Less than 30	155 (13.6)	635 (55.6)	125 (17.6)	404 (56.7)	30 (7)	231 (53.6)
From 30 to 60	369 (32.3)	336 (29.4)	251 (35.2)	198 (27.8)	118 (27.4)	138 (32)
From 60 to 90	320 (28)	108 (9.3)	177 (24.9)	67 (9.4)	143 (33.2)	41 (9.5)
From 90 to 120	150 (13.1)	38 (3.3)	83 (11.7)	27 (3.9)	67 (15.5)	11 (2.6)
From 120 to 180	83 (7.3)	12 (1)	36 (5.1)	8 (1.1)	47 (10.9)	4 (0.9)
More than 180	66 (5.7)	14 (1.2)	40 (5.5)	8 (1.1)	26 (6)	6 (1.4)
**Hours of sleep/week *M* (*SD*)**	9.44 (0.01)	9.66 (0.06)	8.88 (0.06)	9.38 (0.05)	9.39 (0.51)	9.40 (0.07)

During the quarantine, Italian children spent more time using screens (β = 46.56, SE = 0.09, Wald χ^2^ 95% CI [38.37, 56.49], *p* < 0.001), less time daily doing physical activity (β = 0.06, SE = 0.08, Wald χ^2^ 95% CI [0.05, 0.07], *p* < 0.001), and more time sleeping during the week (β = 1.47, SE = 0.04, Wald χ^2^ 95% CI [1.35, 1.61], *p* < 0.001) compared to before the quarantine. Spanish children spent more time using screens (β = 152.69, SE = 0.14, Wald χ^2^ 95% CI [114.36, 203.88], *p* < 0.001), less time daily doing physical activity (β = 0.01, SE = 0.13, Wald χ^2^ 95% CI [0.01, 0.02], *p* < 0.001), and more time sleeping during the week (β = 1.24, SE = 0.07, Wald χ^2^ 95% CI [1.07, 1.42], *p* < 0.01) compared to before quarantine.

Differences between Italy and Spain were found in the use of screens during the quarantine (β = −0.83, SE = 0.13, Wald χ^2^ 95% CI [−1.01, −0.56], *p* < 0.001). During the quarantine, Spanish children tended to spend more time using screens than did Italian children, such that 89.7% of the Spanish children used screens for at least 60 min per day, whereas 81.6% of the Italian children did so. No differences between Spain and Italy in physical activity during the quarantine (β = 0.22, SE = 0.14, Wald χ^2^ 95% CI [−0.05, 0.50], *p* = 0.11) and mean hours of sleep during the week (β = −0.01, SE = 0.09, Wald χ^2^ 95% CI [−0.20, 0.16], *p* = 0.84) were found. All models were adjusted for sociodemographic differences between the samples from Italy and Spain, parental age, and stress.

### Relationship Between Primary Caregivers’ Perception of COVID-19 and Their Children’s Patterns of Use of Screens, Daily Physical Activity, and Hours of Sleep

Spearman correlations indicated that changes in child routines were related to parental perception on how easy it is for the family to live together during the quarantine, parental perception on how serious the situation is, and parental stress. When family coexistence during the quarantine was rated as easier, parents informed that their children tended to spend more time doing physical exercise (ρ = 0.08; *p* = 0.004). For parents who perceived the situation as more serious, their children made more use of screens (ρ = 0.07; *p* = 0.01) during quarantine compared to the rest. Parents with higher levels of stress tended to report that their children made more use of screens (ρ = 0.05; *p* = 0.01), spent less time on physical activity (ρ = −0.10; *p* ≤ 0.001) and slept fewer hours than the rest (ρ = −0.12; *p* ≤ 0.001).

When the Italian sample was selected, changes in child routines were related to parental perception on how serious the situation is and parental stress. Parental perception of how easy coexistence is during quarantine was not related to changes in child habits during home confinement. Parents who perceived the situation as more serious had children who made greater use of screens (ρ = 0.10; *p* = 0.008), compared to the rest. Parents who were more stressed by the situation reported that their children slept fewer hours compared to the rest (ρ = −0.08; *p* = 0.02).

When the Spanish sample was selected, changes in child routines were related to parental perception on how easy it is for family to live together during quarantine and parental stress. When family coexistence during the quarantine was rated as easier, parents informed that their children tended to spend less time using screens (ρ = −0.09; *p* = 0.04), spend more time doing physical exercise (ρ = 0.16; *p* = 0.001), and sleep more hours (ρ = 0.11; *p* = 0.02). Parents who were more stressed by the situation reported that their children spent less time doing physical activity (ρ = −0.22; *p* ≤ 0.001) and slept fewer hours compared to the rest (ρ = −0.08; *p* = 0.02).

## Discussion

This study is the first one developed to examine the psychological impact of the COVID-19 quarantine on children and adolescents of the West. Italy and Spain are two of the countries most affected worldwide by COVID-19, and the emotional well-being of youth needs to be explored to provide parents and caregivers with guidelines to reduce the impact of the quarantine on their children.

Results indicate that 85.7% of the parents reported changes in their children’s emotional state and behaviors during the quarantine. The most frequent symptom was difficulty concentrating, with 76.6% of the parents reporting this symptom. Boredom, irritability, restlessness, nervousness, feelings of loneliness, and being more uneasy and more worried were reported by more than 30% of parents. As expected, and according to the study of Xie et al. (2020) with Chinese children, the COVID-19 confinement also impacts negatively on the Italian and Spanish children’s well-being. Results by country show that Spanish children were more affected than Italian children on most symptoms. Although more research is needed to clarify this finding, the permission given on March 31 by the Italian government to parents to take under-18-year-old youths for a short walk (access to parks, gardens, and all kinds of public spaces is still not allowed) might explain the differences between Spain and Italy. Whereas in Italy, after 3 weeks of home confinement, children were allowed to go out for a short walk close to home accompanied by one adult, in Spain, children could only go outside for a justified reason. Furthermore, many more Italian homes have gardens compared to Spanish homes (52 vs. 18%), so this provided children with the opportunity to be more active, benefiting their mental well-being. These differences in rules and the characteristics of the homes of the two countries may explain why Spanish children showed a worse behavioral and emotional response to the quarantine. Staying at home without the chance to go outside may increase responses of anxiety and other related problems such as sleep problems, physical complaints, and worries and also behavioral problems that involve the family, such arguing with other relatives, maybe because the children need to be more physically active. In contrast, Italian children were perceived by their parents as sadder, and lonely, maybe due to the longer duration of the quarantine in Italy compared to Spain. As expected, routines also changed during the quarantine for the children of both countries, spending more time using screens, spending less time doing physical activity, and sleeping more hours. For instance, during the quarantine, only 14.8% of the youth practice at least 60 min of physical activity, as recommended by the WHO for children between 5 and 17 years old, compared to before the quarantine, when that percentage reached 54.1%. In accordance with a review of studies that observed differences in habits between weekdays and vacation days, the present study found more healthy routines in youth before the quarantine (Brazendale et al., 2017). This finding is also in line with other reflections that warn about the consequences of children’s home confinement, specifically affecting not only their mental well-being but also their weight status and the risk of increasing screen time (Lancet Child Adolescent Health, 2020).

Although only 11.4% of the Italian and Spanish parents informed that family coexistence was difficult or very difficult, most parents perceived the situation due to COVID-19 as serious or very serious, and one-third of the parents reported being stressed or very stressed, with no differences between the two countries. Furthermore, parents’ perception of COVID-19 seems to be related to their children’s psychological symptoms during the quarantine. When family coexistence during the quarantine was more difficult, the situation was more serious, or the level of stress was higher, parents tended to report more emotional problems in their children; specifically, the primary caregiver’s level of stress was related to 25 of the 31 child symptoms. Child routines were also related to the parents’ perception of COVID-19. Specifically, a higher level of parental stress was related to more use of screens, less time of physical activity, and fewer hours of children’s sleep. Different correlation patterns between children’s routines and parental variables were found in Spain and Italy, although parental stress was common in both. Although a causal relationship cannot be confirmed, it is expected that children’s behavioral problems will negatively affect the family climate, and also, parents with a high level of stress may apply more inconsistent discipline with their children.

## Conclusion

COVID-19 quarantine impacts considerably on Italian and Spanish youth’s lives, and most parents perceive changes in their children’s emotional state and behaviors during the quarantine compared to before the quarantine. Spanish children show more emotional and behavioral symptoms compared to Italian children, as reported by their parents, possibly because Italian youth have more opportunities to be more physically active. The emotional and behavioral symptoms of children from both countries seem to be positively related to the parents’ well-being, specifically to their level of stress. On the basis of these findings, we can conclude that children and parents are both affected by such a stressful situation as the quarantine. The present study has some limitations. Although a multi-informant method would be desirable, self-reports were not adequate for the youngest children. As a main objective of this study was to evaluate emotional and behavioral changes in children before and during confinement, assessing for themselves whether they manifested each symptom more or less frequently was not considered adequate for children. Also, as behavioral symptoms were included in the survey (such as arguing with the rest of the family, restlessness, or irritability), it was considered that the parents were better informants than the children. Despite this limitation, this study is the first providing data on the psychological repercussions of the COVID-19 quarantine on children and adolescents from Western countries. Some practical implications can be derived from the results. First, the obtained data could help governments to decide the confinement rules to apply to children to preserve their mental health (such as considering the importance of going out for daily walks). Second, our results could guide parents and caregivers. For example, as being worried when parents leave home is common in children under confinement, clear and age-appropriate information could reduce their uncertainty and concerns. Also, since the greater the stress of the parents, the more symptoms in the children, not expressing frequent concerns in front of their children could help parents to protect their mental health. Finally, professionals should be alert to the more common psychological responses of youth to detect the need for intervention as early as possible. Vulnerable children and adolescents, including those with risk factors, should receive special attention (Espada et al., 2020). In line with this, as Liu et al. (2020) recommended, receiving training to detect children’s psychological problems should be a main objective for pediatric health-care workers. Also, programs focused on providing children with psychosocial skills could help them cope with the COVID-19 situation (Orgilés et al., 2020).

## Data Availability Statement

The data that support the findings of this study are available from the corresponding author upon reasonable request. Requests to access the datasets should be directed to MO, morgiles@umh.es.

## Ethics Statement

The studies involving human participants were reviewed and approved by the Ethics Board of the Miguel Hernández University of Elche. Written informed consent to participate in this study was provided by the participants’ legal guardian/next of kin.

## Author Contributions

MO designed the study and the survey. AM managed and analyzed the data. ED designed the Italian survey and collected the data. CM participated in the Italian survey adaptation and collected data. JE designed this study and wrote the draft of this article. All the authors reviewed the draft and contributed to the final version of the manuscript.

## Conflict of Interest

The authors declare that the research was conducted in the absence of any commercial or financial relationships that could be construed as a potential conflict of interest.
